# Constraining the Range and Variation of Lithospheric Net Rotation Using Geodynamic Modeling

**DOI:** 10.1029/2021JB022057

**Published:** 2021-10-19

**Authors:** Suzanne Atkins, Nicolas Coltice

**Affiliations:** ^1^ Laboratoire de Géologie CNRS‐École Normale Supérieure‐PSL University Paris France

**Keywords:** net rotation, geodynamics, mantle, plate reconstruction, lithosphere

## Abstract

Lithospheric net rotation (LNR) is the movement of the lithosphere as a solid body with respect to the mantle. Separating the signal of LNR from plate tectonic motion is therefore an important factor in producing absolute plate motion models. Net rotation is difficult to constrain because of uncertainties in geological data and outstanding questions about the stability of the mantle plumes used as a reference frame. We use mantle convection simulations to investigate the controlling factors for the magnitude of LNR and to find the statistical predictability of LNR in a fully self‐consistent convective system. We find that high lateral viscosity variations are required to produce Earth‐like values of LNR. When the temperature dependence of viscosity is lower, and therefore slabs are softer, other factors such as the presence of continents and a viscosity gradient at the transition zone are also important for determining the magnitude of net rotation. We find that, as an emergent property of the chaotic mantle convection system, the evolution of LNR is too complicated to predict in our models. However, we find that the range of LNR within the simulations follows a Gaussian distribution, with a correlation time of 5 Myr. The LNR from the models needs to be sampled for around 50 Myr to produce a fully Gaussian distribution. This implies, that within the time frames considered for absolute plate motion reconstructions, LNR can be treated as a Gaussian variable. This provides a new geodynamic constraint for absolute plate motion reconstructions.

## Key Points

- Net rotation is an emergent property of a chaotic system and is difficult to predict, but the range and distribution can be constrained
- Large lateral viscosity contrasts are required for an Earth‐like magnitude of net rotation in geodynamic models
- Net rotation follows a Gaussian distribution over timescales greater than 50 Myr with a correlation time of 5 Myr

## 1 Introduction

Quantifying the net rotation of the Earth's lithosphere remains an unsolved problem in geodynamics and plate tectonic reconstructions. Net rotation is the movement of the entire lithosphere as a solid body with respect to the Earth's mantle. It is therefore one component of the motion recorded with respect to the Earth's magnetic dipole, preserved in the palaeomagnetism of rocks. However, three other components of motion are also preserved in the palaeomagnetism (Jurdy, 1981): movement of individual plates (continental drift); rigid‐body rotation of the lithosphere and mantle with respect to the Earth's spin axis (true polar wander; Duncan & Richards, 1991; Steinberger & Torsvik, 2008); and movement of the Earth's magnetic dipole relative to the Earth's spin axis. The contribution of these processes to palaeomagnetic data cannot be separated. In order to study any one of these components, for example when building absolute plate motion models, assumptions for the others must be made in order to reconstruct the movement of the lithosphere through geological history (Coltice et al., 2017; Tetley et al., 2019).

The lithospheric net rotation (LNR) estimates require a stable reference point in the mantle. Mantle plumes and hotspots are generally used, as these are considered to have been roughly stable for the last few tens of millions of years (Duncan & Richards, 1991; Maher et al., 2015; Morgan, 1971; Wilson, 1963), although there is disagreement about which, if any, plumes should be considered stable (Bono et al., 2019; Doubrovine & Tarduno, 2008; O'Neill et al., 2005), especially since geodynamic modeling suggests that plumes can move rapidly (Arnould et al., 2020). Ridge spreading direction, preserved in the mantle as anisotropy may also be used to produce reference frames for plate reconstructions because in certain tectonic settings it aligns with absolute plate motion (Williams et al., 2016).

Absolute plate motion reconstruction models all produce some LNR, although the magnitude varies depending on the reconstruction. This variation is partly due to differences in the choice of reference frame used. The creation of absolute plate motion models requires high‐quality, widespread palaeomagnetic data which is only available for the relatively recent geological past. The further back in geological time one goes, the sparser and more unreliable the palaeomagnetic data becomes, reducing the certainty with which absolute plate motions can be reconstructed, and therefore estimates of LNR also become more unreliable.

The range of LNR values predicted by absolute plate motion models goes from around 0.13°/Myr (Torsvik et al., 2010) to 0.44°/Myr (Gripp & Gordon, 2002) when a deep hotspot reference frame is used, potentially increasing to >1°/Myr if shallow reference frames are used (Doglioni et al., 2015), or as low as 0.11°/Myr when the models are setup to explicitly minimize LNR (Tetley et al., 2019). Studies of seismic anisotropy, which can be used as a proxy for shearing between the lithosphere and the mantle suggest amplitudes of 0.2–0.3°/Myr (Becker et al., 2008, 2014; Conrad & Behn, 2010; Kreemer, 2009), which are within the range proposed by plate models. In the absence of constraints on LNR, one solution is to minimize LNR at all times in plate motion reconstructions (Tetley et al., 2019). However, these models generally aim to fit geological and palaeomagnetic data but are not constrained by the physical laws that control geodynamic flow.

In order to improve absolute plate reconstructions and extend them further into the past, we need to know how much and over what time scales LNR can reasonably vary. Geodynamic modeling lets us explore the ranges and timescales of variation, allowing us to assess whether the LNR predicted by plate reconstruction models is geophysically reasonable. Ideally, we would also be able to identify tectonic situations which lead to high or low net rotation. We could then make an estimate of the likely LNR in a particular global tectonic configuration even if there is insufficient palaeomagnetic data to constrain a reconstruction well. In order to do this, we investigate the origins of LNR in mantle convection models.

The movements of the convecting mantle can be decomposed into poloidal motions, the up‐ and downwellings caused by buoyancy differences, and toroidal motions, the origin of which is generally attributed to lateral viscosity variation (Bercovici et al., 2000). Net rotation of the lithosphere is the degree 1 of the toroidal component of velocity. In this study, we use two‐dimensional simulations. The poloidal motions and degree 1 toroidal component (LNR) can be studied in 2D, depending on the geometry of the model. Although we lose the higher orders of the toroidal component by using 2D models, the computational saving compared to using expensive 3D models means we can investigate a greater range of parameter combinations to constrain some basic relationships between convection parameters and net rotation. We use the mantle convection code StagYY (Tackley, 2008) with spherical annulus geometry (Hernlund & Tackley, 2008). Unlike in 2D models which use an axisymmetric geometry, net rotation of the lithosphere is possible in a spherical annulus. Similar studies have also been performed using a 2D cylindrical geometry (Gérault et al., 2012), but spherical annulus geometry has the advantage that velocities are closer to those expected in a full 3D simulation (Hernlund & Tackley, 2008).

The convection models allow us to investigate which parameters control the magnitude and variability of LNR. It has long been recognized that lateral viscosity variations are required to produce net rotation (Alisic et al., 2012; Becker, 2006; Gérault et al., 2012; Ricard et al., 1991; Zhong, 2001), Continents provide a strong lateral viscosity variation (Becker, 2006; Rudolph & Zhong, 2014; Zhong, 2001), enhanced if they have deep roots, as do temperature and stress‐dependent viscosity laws (Alisic et al., 2012). Deep mantle viscosity structure affects the speed of plate motion, by determining the sinking rate of slabs. A viscosity increase at the 660 km transition zone may slow or stop slab penetration into the deep mantle (Conrad & Lithgow‐Bertelloni, 2002), while increasing temperature dependence of viscosity in the lower mantle increases the viscous drag around the slab tip, slowing its sinking (Alisic et al., 2012). Other factors that have been proposed to affect net rotation include the position of oceanic ridges, trench migration, slab properties, and a weak asthenospheric layer. Gérault et al. (2012) investigated these factors in 2D convection models and found that asymmetric slab dips and ridge positions promoted net rotation. However, geodynamical studies tend to find NR rates which lie at the lower end of the ranges proposed by plate tectonic models, generally less than 0.15°/Myr (Becker, 2006; Ricard et al., 1991; Zhong, 2001), except the models of Alisic et al. (2012), which have NR values up to around 0.25°/Myr. The earlier geodynamic modeling studies did not use highly temperature‐dependent viscosity. Slabs in the mantle could therefore be relatively soft. In models that have larger lateral viscosity variations, such as those used in this study and Alisic et al. (2012), slabs remain stiff, and values achieved for net rotation are in line with those inferred for Earth.

In this work, we study net rotation in evolving mantle models. Unlike previous studies, we have both high lateral viscosity variation, and therefore stiff slabs in the mantle, and continuously evolving mantle structures. Our models are neither initialized nor constrained by the imposition of present day observations of the surface or mantle structure. This allows us to investigate how net rotation evolves as a statistical and emergent property of a fully self‐consistent mantle system, where all tectonic behavior arises naturally from physical laws, rather than relying on the imposition of surface forcing. We can therefore study the constraints on the magnitude and statistical predictability of LNR in our models, independent of uncertainties and inconsistencies arising from the geological record.

We perform a parameter space search in order to more fully investigate the effects of different geodynamical properties on the magnitude and variation of net rotation. This gives us a statistical way in which we can constrain the net rotation of the Earth's lithosphere for use in absolute plate motion models. We vary a variety of convection parameters that have been identified in previous studies to have effects on the magnitude of net rotation. The models we use can include continents (although not all of our models do) and they develop self‐organizing plate tectonics with spreading ridges and subduction zones. In agreement with previous studies, we find that changing the viscosity, especially the parameters that increase the lateral variation of viscosity, increases the magnitude of lithospheric net rotation. For models with highly temperature‐dependent viscosity and therefore stiff slabs, we achieve ranges of LNR within the published ranges for Earth. Our large viscosity contrasts mean that changing the presence and thickness of continents makes little difference to LNR, because the presence of stiff slabs in the mantle creates a much greater viscosity contrast than the continents, in contrast to previous studies.

## 2 Methods

Our convection models are incompressible and were run using the geodynamical code StagYY with a 2D spherical annulus geometry (Hernlund & Tackley, 2008). The resolution is 1,152 cells azimuthally by 128 radially, with $1.6\times 10^{6}$ Lagrangian tracers. The surface and core‐mantle boundaries both have free‐slip conditions. Where present, two continents are initialized to cover 30% of the surface. They are stiffer and more buoyant than the upper mantle and lithosphere to ensure that they remain at the surface. They have lower yield stress margins, allowing them to form a single supercontinent and subsequently break up. The mantle is initialized with a layer of denser primordial material at the core‐mantle boundary. There is internal heating, but this does not decay with time. We, therefore run the models until they reach a stable whole mantle mean temperature, at which point we judge them to have entered a statistically stable convective state. Once the models have reached a statistical steady state, we run them for at least a further 600 time steps. We use this stable phase of the run for all of our subsequent analyses.

We use a Rayleigh number ($Ra_{0}$) of $10^{6}$, where:

(1) $$Ra_{0}=\frac{\alpha_{0}\rho_{0}g\Delta TD^{3}}{\kappa_{0}\eta_{ref}}=10^{6}$$

$\alpha_{0}$ is the surface thermal expansivity, $\rho_{0}$ is reference density, g gravitational acceleration, ΔT is the difference in temperature between top and bottom, D is mantle thickness, $\eta_{ref}$ is reference viscosity and $\kappa_{0}$ is thermal diffusivity.

We investigate the effects of eight parameters on lithospheric net rotation (Table 1). Details on individual models can be found in Appendix A. Viscosity activation energy ($E_{a}$) and viscosity activation volume ($V_{a}$) determine the response of viscosity, η, to pressure and temperature:

(2) $$\eta =\eta_{0}\exp\left(\frac{E_{a}+V_{a}P}{RT}\right),$$

where P is pressure, R is the gas constant and T is temperature. $\eta_{0}$ is a scaling prefactor used to obtain a volumetrically averaged viscosity equal to $\eta_{ref}$ for an isothermal mantle of potential temperature 0.64 in dimensionless units. After running a calculation, the maximum viscosity is at the surface and the minimum in most cases in the modeled asthenosphere where the temperature is close to 0.75 in dimensionless units. In some models, we include a viscosity jump between the upper and lower mantle by a factor of 30. Viscosity is restricted to between a non‐dimensional range of $10^{-6}$–$10^{4}$. We use an oceanic yield stress to determine the strength of the lithosphere of between 2 $\times \;10^{2}$ and 2 $\times \;10^{6}$, although the majority of simulations have yield stress 2 $\times \;10^{4}$. The basal temperature, which models heating from the core, has a constant non‐dimensional value of 1.12. We also change the internal mantle heating rate. Equivalent dimensional values are given in Table 1.

**Table 1:** Input Parameters Used for Convection Models, With Fixed Parameters First

Parameter	Non‐dimensional values	Dimensional equivalents
Surface temperature	0.12	255 K
Basal temperature	1.12	2,390 K
Mantle thickness (D)	1	2,890 km
Thermal conductivity	1	3.2 W $m^{-1}\;K^{-1}$
Reference thermal expansivity ($\alpha_{0}$)	1	5 $\times \;10^{-5}K^{-1}$
Reference density ($\rho_{0}$)	1	4,300 kg $m^{-3}$
Gravity acceleration (g)	1	9.81 m
Reference diffusivity ($\kappa_{0}$)	1	$10^{-6}\;m^{2}\;s^{-1}$
Reference viscosity ($\eta_{ref}$)	1	$10^{23}$ Pa s
Minimum and maximum viscosity cut‐off	$10^{-6}$ – $10^{4}$	$10^{17}$–$10^{27}$ Pa s
Continent viscosity contrast	10–1,000	Factor
Continent density excess	Buoyancy ratio −0.6	−275 kg $m^{-3}$
Primordial density excess	Buoyancy ratio 0.25	114 kg $m^{-3}$
Viscosity activation energy ($E_{a}$)	0–12	0–213 kJ ${mol}^{-1}$
Viscosity activation volume ($V_{a}$)	0–12	0–6 ${cm}^{3}\;{mol}^{-1}$
Transition zone viscosity jump	1 or 30	Factor
Initial thickness of primordial layer	0.02–0.25	57.8–722.5 km
Primordial viscosity contrast	1–10	Factor
Radiogenic heating	25–100	4.7–18.9 $\times \;10^{-12}$ W ${kg}^{-1}$
Oceanic yield stress	$2\times 10^{2}$ – $2\times 10^{6}$	2.4–24,000 MPa
Continent thickness	0–0.2076	0–600 km
Continent yield stress	$10^{8}$ – $10^{14}$	1.2 $\times 10^{6}$–1.2 $\times 10^{12}$ MPa

*Note*. The models are non‐dimensional, but we provide dimensional equivalents for ease of comparison with other research. See Appendix A for the details of individual models.

The initial layer of dense primordial material at the base of the mantle changes in thickness and viscosity between models, as does the thickness of the buoyant, stiff continents. The continents have a weaker rim to allow them to rift, with a yield stress of $1\times 10^{8}$ and viscosity factor 10 at the outside, increasing to $1\times 10^{14}$ and viscosity factor 1,000 at the center. Some models have no continents.

The spherical annulus geometry we use was developed by Hernlund and Tackley (2008), which reduces a 3D coordinate system to 2D by fixing the dimension of variables perpendicular to the annulus so that there is no variation in this axis. The velocity and gravity vectors also have no components in this neglected dimension. For net rotation, this means that only degree 1 toroidal motion is possible because higher degrees require variations in velocity perpendicular to the slice. Unlike when modeling the mantle using an axisymmetrical geometry, degree 1 net rotation is possible in spherical annulus geometry because it is a laterally uniform equatorial slice. In contrast, the axisymmetric system has internal impermeable reflecting boundaries at the poles, preventing any toroidal velocity component.

In order to solve the Navier‐Stokes equations, it is necessary to define a reference frame for velocity. There are different ways to define the reference frame. StagYY uses a no surface net rotation reference frame to calculate the velocity at each point. Technically speaking, a numerical solution is obtained in an arbitrary reference frame, and a global solid rotation is imposed to move this solution back to the no surface net rotation reference frame. We then post‐process the models to find surface velocity and rotation in a no mean mantle net rotation frame, in a similar way to Zhong (2001). To transfer the velocity field to a no mean mantle net rotation frame, we first compute the mean mantle net rotation in the no surface net rotation frame by summing the angular velocity, ω for each azimuthal and radial element, θ and r, in the model:

(3) $$\text{net}\;\text{mantle}\;\text{rotation}=\frac{\omega_{\theta r}V_{\theta r}}{V}$$

proportional to its volume, $V_{\theta r}$, summed over θ and r and divided by total volume V, to give the total mantle net rotation. We then remove that component of the velocity field, leaving the kinematic information about net rotation.

## 3 Results

### 3.1 Convection Parameters and Net Rotation

In this study, we vary eight convection parameters, over 110 models. The only parameter that makes a clear difference when considered alone is the activation energy of viscosity, $E_{a}$ (Equation 2), which can change LNR by up to two orders of magnitude in models where all other parameters are the same. This parameter changes the temperature dependence of viscosity. A higher value of $E_{a}$ leads to greater lateral viscosity variations, particularly increasing the stiffness of slabs in the mantle, and therefore larger lithospheric net rotation (LNR). This result is in line with the results of previous studies (Becker, 2006; Alisic et al., 2012), however, we use much greater viscosity variations than in Becker (2006), with lateral viscosity variations of up to seven orders of magnitude in the upper mantle when $E_{a}=8$.

**Figure 1:**
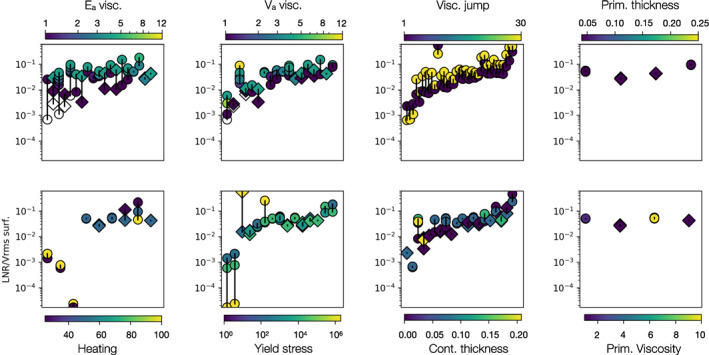
Net rotation for sets of sibling models. Each set, joined by vertical black lines, has the same values for 7 out of 8 convection parameters. They are colored by the one varying parameter. The sets are ordered by increasing $E_{a}$ along the *x*‐axis, with the exception of the $E_{a}$ case (top left), where models have no order along the *x*‐axis. Models marked by circles have a viscosity jump, diamonds have no viscosity jump. White points have $E_{a}$ or $V_{a}$ = 0.

The dominant effect of $E_{a}$ (Equation 2) hides the relationship between the other parameters and LNR. We, therefore, consider sibling sets of models. Within each sibling set, the models have the same values for seven out of eight model parameters. Those seven shared parameters are different for each set. This allows us to isolate the effects of each parameter and how the effect changes in conjunction with other parameters. Due to the expense of running the convection models, we have not covered the parameter space with an even grid of models therefore some parameters have more sibling sets than others. Figure 1 shows the sibling sets, ordered by increasing $E_{a}$. We present the variations caused by each parameter when the LNR is divided by the RMS surface velocity. We do this because the convective vigor varies significantly between models. By scaling by the RMS surface velocity, we effectively view the models as if they all had the same convective vigor. The pattern is the same if the raw LNR is used. From these sibling sets of models, we observe relationships between LNR and several convection parameters.

#### 3.1.1 Viscosity Activation Energy

An increase in viscosity activation energy, $E_{a}$, always leads to an increase in LNR, regardless of the other parameters used. Increasing $E_{a}$ can produce nearly two orders of magnitude increase in LNR. This huge change is because going from $E_{a}=0$ to $E_{a}=1$ adds lateral viscosity variations into the mantle. Subsequent increases produce a more incremental effect, but in general doubling $E_{a}$ leads to an approximate doubling of LNR.

#### 3.1.2 Viscosity Activation Volume

An increase in viscosity activation volume, $V_{a}$ (Equation 2), generally, but not always, leads to an increase in LNR. The increase can be up to 1 order of magnitude for a 5‐fold increase in $V_{a}$, although for most parameter combinations, a 3–5‐fold increase in $V_{a}$ produces around a doubling of LNR. The only sibling set where $V_{a}$ does not increase LNR has very thick continents and $E_{a}$ = 1. The two models have LNR with the same order of magnitude, therefore we attribute the decrease in LNR with increased $V_{a}$ to the chaotic nature of the evolution of LNR and suggest that the models are nearly statistically identical. Increasing $V_{a}$ also increases temperature dependence of viscosity, although less directly than $E_{a}$ (see Equation 2). It has a correspondingly smaller effect on LNR, with magnitude variation independent of $E_{a}$.

#### 3.1.3 Viscosity Jump

A factor 30 increase in viscosity at the transition zone almost always produces an increase in LNR. The implementation of the viscosity jump in our models effectively reduces the viscosity of the upper mantle compared to models without a jump, whilst the lower mantle has comparable viscosity in all models. The addition of a viscosity jump increases LNR between comparable models by one to two orders of magnitude, with a greater increase in models with lower $E_{a}$. In models with higher $E_{a}$, a reduction in viscosity by a factor 30 produces a much less pronounced increase in lateral viscosity variations because the thermal effects on viscosity are much greater than ×30.

#### 3.1.4 Continent Presence and Thickness

At low values of $E_{a}$, adding continents increases LNR by up to an order of magnitude. However, with increasing $E_{a}$, this relationship breaks down. The thickness of the continents, once they are present (thickness > 0), makes very little difference to LNR at any $E_{a}$. Previous studies found that both the presence and thickness of continents (Becker, 2006; Zhong, 2001) had an impact on LNR rates. However, these studies have much lower lateral viscosity variations (e.g., lateral viscosity variation of around two orders of magnitude in Becker, 2006), whilst ours have lateral viscosity variations of over seven orders of magnitude. When slabs can produce such a large lateral viscosity variation in the mid‐mantle, the smaller viscosity variation produced by stiff continents at the surface is of secondary importance. Our low $E_{a}$ models show similar results to previous studies, because they share similar rheological properties.

No other parameter makes a reliable difference to LNR. Very high yield stress causes the models to stagnate. This tends to lead models to have relative time‐invariant values of net rotation. In some cases, the LNR remains around zero, but it can also lead to moderately high magnitude uni‐directional net rotation. These simulations, therefore, appear to have higher magnitudes of LNR/$V_{rms}$ when the stable runtime average is plotted in Figure 1, but are in fact relatively inactive compared to the other models. Changing the thickness and viscosity of the layer of dense material at the base of the mantle makes no difference to the LNR. Increasing the internal heating through the decay of radioactive elements also makes no difference to the magnitude LNR, despite this affecting the convective vigor. Variation within each sibling set for these parameters is due to the chaotic nature of mantle convection, meaning that a small variation can change the convection patterns. With longer run times, we expect these model sets to converge to have stable, near‐identical average rates of LNR.

### 3.2 Viscosity and LNR

**Figure 2:**
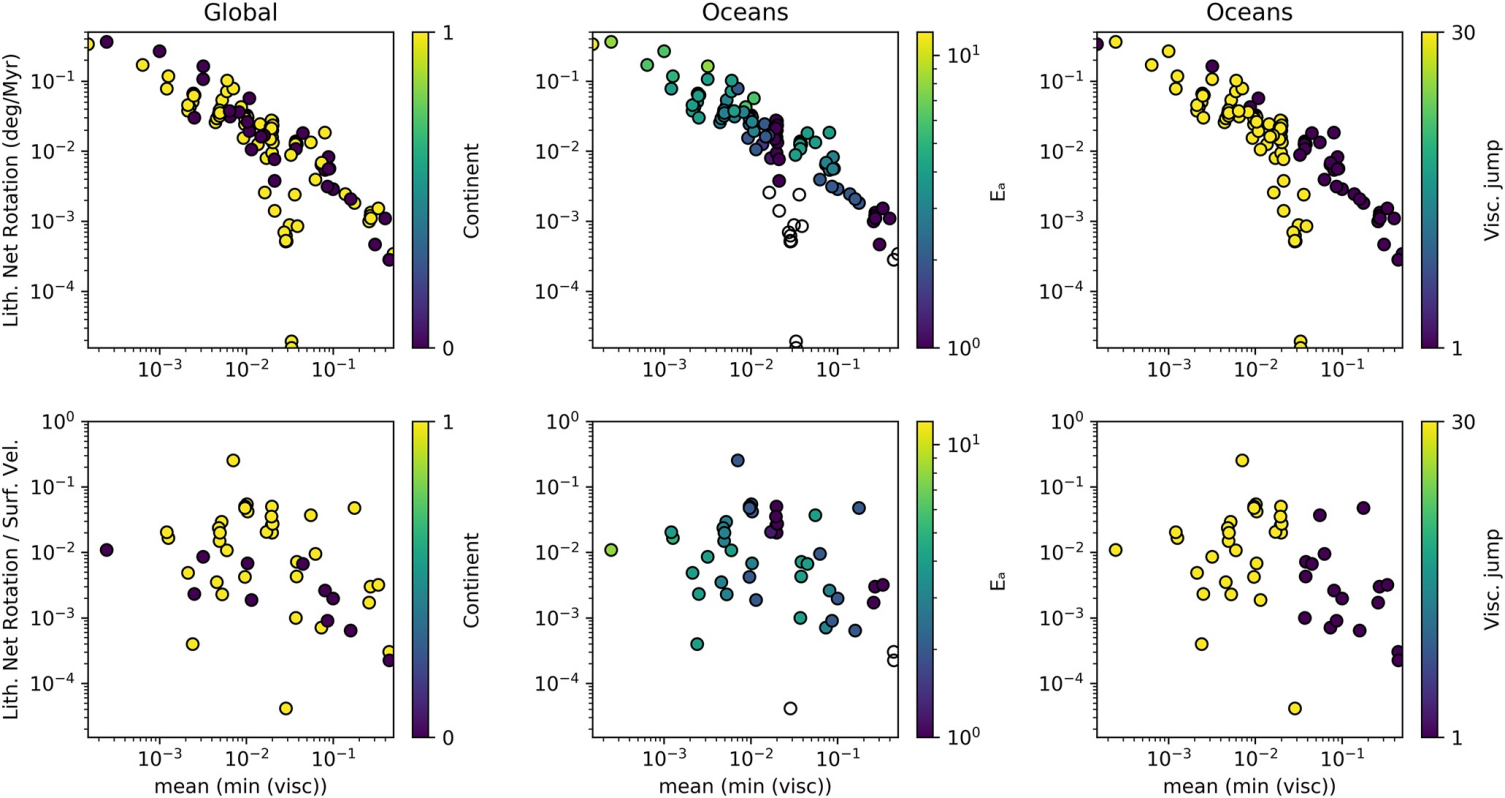
Relationship between average minimum viscosity in the upper mantle and lithospheric net rotation (LNR). The top row is the simple LNR. The bottom row shows LNR scaled by the surface Vrms in order to remove the effects of convective vigor. On the left: the minimum viscosity at each time step, averaged over the life of the model, colored by whether the model has continents. Center: average minimum viscosity below oceans, colored by $E_{a}$. Right: average minimum viscosity under oceans, colored by the magnitude of the viscosity jump at the transition zone. White points have $E_{a}$ or $V_{a}$ = 0.

In Section 3.1, four convection parameters were identified that had a discernible effect on lithospheric net rotation. Three of these parameters directly control the viscosity structure of the models. The fourth, continental lithosphere, introduces large viscosity variations at the surface. The relationship between viscosity and LNR becomes much more obvious when just the upper mantle is considered (Figure 2). In the lithospheric upper mantle (at depth 0.04, equivalent to 115 km), we see a log‐linear relationship between the minimum viscosity at each time step, averaged over the models' run, and the root mean squared LNR. We use the minimum viscosity because at 115 km the continental roots all have the same maximum viscosity. The continents, therefore bias the mean viscosity, meaning that models with and without continents cannot be compared. We show the same results twice, first with LNR and then with the LNR scaled by the RMS of the surface velocity.

The presence of continents makes no significant difference to the relationship between viscosity and LNR. The relationship is dependent on the minimum viscosity under the oceans, with the relationship being almost identical when the continental lithosphere is excluded (middle and right columns, Figure 2) because the minimum viscosity almost always occurs under the oceans. The minimum viscosity, and therefore the net rotation, is dependent on the viscosity parameters chosen in the model, namely the presence of a viscosity jump and the activation energy of viscosity, which determines the sensitivity of viscosity to temperature variations. However, when LNR is scaled by Vrms, the relationship weakens. This is expected because much of LNR magnitude is determined by the vigor of convection, which is a function of viscosity.

### 3.3 LNR Variation With Time

The results presented so far were all for the RMS value of net rotation over the runtime of the model, with some correlations between parameters and LNR found when we consider the full suite of models. Whilst this gives a statistical average, it does not give details about the expected value at any time step nor how the rotation varies within the model. For absolute plate motion models, estimates of how much LNR varies with time are required. We therefore investigate if particular tectonic and convective configurations can be linked to LNR magnitude and the timescales over which LNR varies.

Visual inspection of the evolution of individual models does not yield any clear relationship between geodynamic patterns and changes in net rotation. Several models change from having regular supercontinent accretion and break‐up cycles to having permanent supercontinents. In most of these models, but not all, the phase where the continents regularly disperse and converge is associated with lower LNR. The RMS surface velocity does not change when the models switch to a single, stable supercontinent, therefore the increase LNR is not caused by an increase in convective vigor when a single supercontinent dominates. It seems that the frequent change in direction of continent motion as they converge and dispersal constraints the LNR, probably by maintaining higher degrees of symmetry in the model. However, during this alternating phase, the net rotation does not increase when the continents have come together.

**Figure 3:**
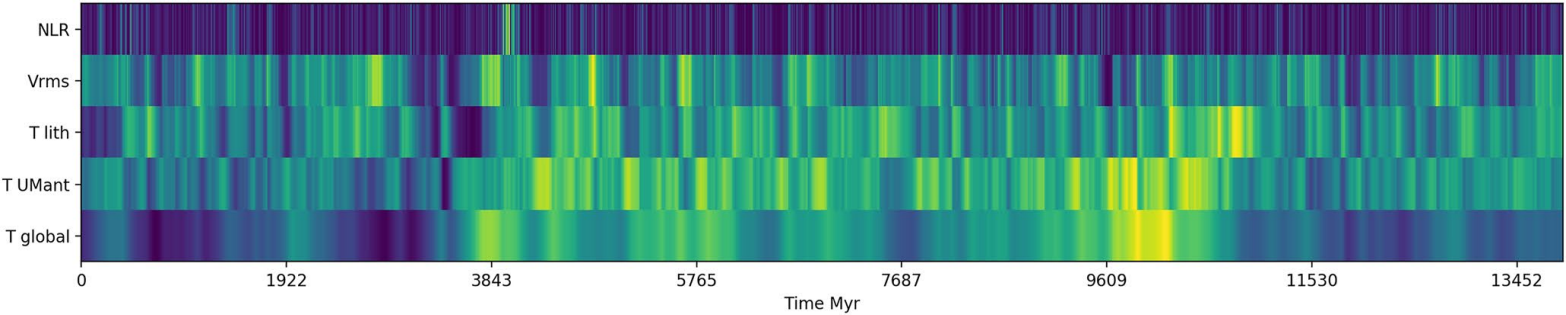
Variations in lithospheric net rotation, surface root mean square velocity, mean lithospheric temperature, mean upper mantle temperature, and mean whole mantle temperature in model 12. The color scale is the minimum (blue) to maximum (yellow) of each parameter. We do not show scaling because we want to highlight the timescales of magnitude change, rather than the magnitudes themselves. Snapshots from this model are shown in Figure 4.

The majority of models do not have supercontinent cycles. The continents converge during the pre‐stabilization phase before analysis starts and do not subsequently diverge. In these models, there is no clear link between geodynamic processes and LNR. We present model 12 (see Appendix A for details) as a representative model, having mid‐range viscosity parameters ($E_{a}=V_{a}=3.0$) and continents. The maximum LNR is 0.184°/Myr, although for most of its life the LNR does not exceed 0.05°/Myr. Figure 3 shows the variation of several convection metrics. We see that variations in temperature and convective vigor are not clearly correlated with LNR. LNR varies on much shorter timescales than temperature. Surface velocity also varies on shorter timescales than temperature, but peaks in surface velocity are not correlated with peaks in LNR.

**Figure 4:**
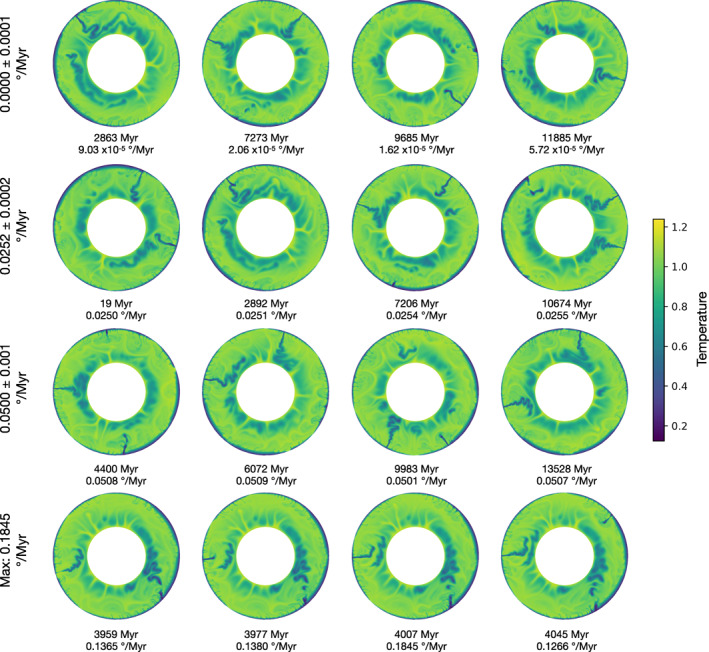
Snapshots from model 12 showing the temperature structure of the mantle, all using the same non‐dimensional color scale. The rows all have similar values of net rotation, but the snapshots are taken at least 500 Myr apart, apart from the bottom row which shows snapshots from the peak of lithospheric net rotation in this model. The model has $E_{a}$ = $V_{a}$ = 3.0, a transition zone viscosity jump and continents.

Sixteen snapshots of the temperature of the mantle from model 12 are shown in Figure 4. They are ordered according to LNR, with low, medium, and high values, and samples from the single very large peak in LNR at 4,000 Myr. The snapshots in the first three groups are all far enough apart in age to be uncorrelated. There is no clear difference between snapshots with low or high LNR. They all have active subduction zones with slabs extending to the lower mantle, with at least one in each category having a slab adjacent to a continent. All have at least three ridges with the hot, thin oceanic lithosphere, and they all have upwellings and slab remnants in the lower mantle. The last row are snapshots taken from the peak of net rotation at 4,000 Myr, which is significantly above average for the model. At the beginning of the peak (3,959 Myr), both slabs have broken off, disconnecting the lithosphere from the mantle. This could explain why the lithosphere is free to rotate. However, we see this elsewhere in the model's history without a corresponding peak in LNR. The cold slab remnants in the lower mantle are also asymmetrically distributed, but we see similar uneven distribution in the top left snapshot with very low LNR. What has caused this peak is therefore unclear. This model is representative in demonstrating the difficulty of finding a relationship between mantle structure and LNR. We have investigated a variety of metrics from the models, using viscosity, temperature, composition, radial, and azimuthal velocity, to which we have applied various image and signal processing methods, but we have been unable to find a robust and reliable predictor of temporal variations in LNR.

Because we are unable to find a clear relationship between mantle structure and LNR by simply inspecting our models, we turn to statistical descriptions of mantle structure in order to describe how LNR changes as models convect. This allows us to make generalized descriptions of the models' behavior, even if we cannot say precisely what the LNR of the models will be at a given time.

The first observation is that, for all of the models, the variation in LNR is Gaussian about the model mean, with standard deviation close to the RMS of LNR. Having established that the distribution of values is predictable if the ${LNR}_{\text{RMS}}$ is known and that ${LNR}_{\text{RMS}}$ is dependent on the minimum asthenospheric viscosity and the surface velocity from Figure 2, we can estimate the range over which LNR varies within our models.

We then investigate how the 1D profiles of temperature and viscosity are correlated with LNR during the stable runtime of each individual model. We can see if particular changes within a model tend to be linked to a change in net rotation, for example, a decrease in temperature, generally caused by increased subduction. This gives us a method to identify convection features that may control LNR, even if they are not clear from inspection of the models. We use the Spearman rank correlation coefficient to correlate changes in the 1D mantle structure during the stable runtime of our models with changes in LNR. This measures the monotonic relationship between two parameters and has the advantage that the relationship does not have to be linear in order to produce a high correlation. The Spearman rank correlation coefficient is simply the Pearson correlation coefficient between the rank variables of two parameters, x and y, and can be calculated by:

(4) $$r_{s}=1-\frac{6\sum {\left(rx_{i}-ry_{i}\right)}^{2}}{n^{3}-n}$$

where $rx_{i}$ and $ry_{i}$ are the ranks of x and y values respectively, ranging from 1 to n.

**Figure 5:**
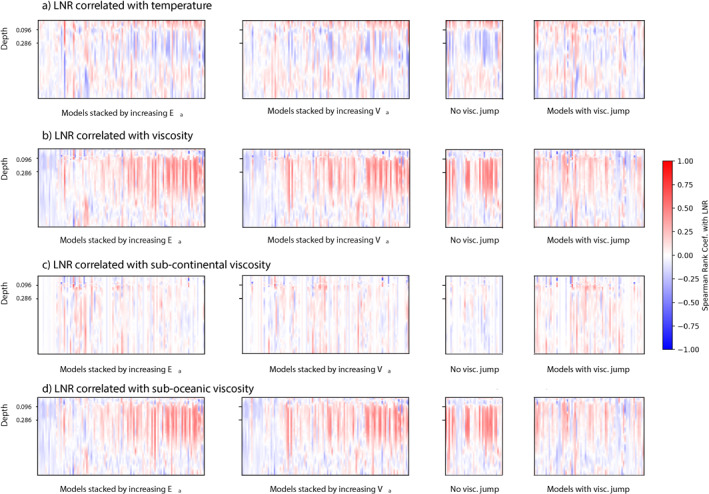
Spearman rank correlation coefficient between lithospheric net rotation and four different radial model observations: (a) 1D temperature structure, (b) 1D viscosity structure, (c) 1D sub‐continental viscosity, (d) 1D sub‐oceanic viscosity—over the stable runtime of the models. The same data is plotted in each column, but models are ordered along the x‐axis according to $E_{a}$, $V_{a}$ and then separated into two grids by the presence of a viscosity jump, where the models are ordered by $E_{a}$.

The correlation between the magnitude of LNR and 1D profiles of temperature and viscosity is calculated separately for each individual model, and the stacked results for all the models are shown in Figure 5. Positive correlations mean that magnitude of LNR increases with the parameter in question, negative that one increases as the other decreases. There is no feature that is universally correlated with LNR across all models. However, when individual models are considered, some relevant correlations emerge.

#### 3.3.1 Correlation of Thermal Profile With LNR

Each set of 1D correlation profiles are stacked according to the convection properties of the models, with increasing values of $E_{a}$ and $V_{a}$ in the first two columns and then the models are separated depending on if they have a viscosity jump in the last two columns. The first row shows the correlation between thermal structure and LNR for each model. The correlation between thermal structure and LNR varies only slightly depending on the convection parameters used. Near the surface, the thermal structure is positively correlated with LNR for depths above 0.096, which corresponds to the base of the continents in the majority of models. The correlation is weak, below 0.5 in all cases. Going deeper, the upper mantle temperature, below the lithosphere, is controlled by the changing balance between cold down‐going slabs and hot upwelling material. Colder upper mantle has a weak correlation with increased LNR because a more mobile surface produces more slabs which cool the upper mantle. Below this, no pattern in correlation emerges. Models with low $E_{a}$ and $V_{a}$ show very little correlation between thermal structure and LNR, although this is not surprising since these simulations have very low LNR in the first place. Simulations without a viscosity jump show a greater change in correlation direction below the continents because they have a higher upper mantle viscosity, making them more temperature‐sensitive. In simulations with a transition zone viscosity jump, fewer than half show any change in correlation around the transition zone (depth 0.286).

#### 3.3.2 Correlation of Viscosity Profile With LNR

The strength of the correlation between the 1D viscosity profile and LNR is very clearly a function of the viscosity parameters chosen. Increasing $E_{a}$ and $V_{a}$ both increase the positive correlation in the mid‐mantle. These parameters enhance the viscosity contrast of slabs, highlighting that at times when there are more slabs in the mid‐mantle, net rotation is often stronger. The effects of a fast‐moving asthenosphere are not visible in the viscosity correlation. This is because the sensitivity of viscosity to temperature is somewhat lower at lower pressures. The correlation is also stronger in simulations without a viscosity jump, because these have higher upper mantle viscosity. We also consider the viscosity profile below the oceans and continents separately. Where present, the continents cover 30% of the surface. The sub‐oceanic mantle makes up the majority and it is therefore unsurprising that the sub‐oceanic correlations are more similar to the global viscosity correlations. The sub‐continental mantle shows very little correlation with NR in any simulation, although we do see a slight increase in correlation immediately below the continents (depth 0.096) in some simulations. This is again related to surface mobility ‐ stationary continents insulate the upper mantle, reducing viscosity. More mobile continents move over colder areas and so will have slightly higher subcontinental viscosity, which correlates with higher mobility and net rotation. The positive mid‐mantle correlations in high $E_{a}$ and $V_{a}$ cases continues for sub‐oceanic profiles. Most slabs are subducted in sub‐oceanic mantle so this is to be expected. The effects of the continents can be seen with an increase in correlation strength at a depth of 0.096. Above this depth, the contribution of continents limits the correlation between viscosity and LNR.

**Figure 6:**
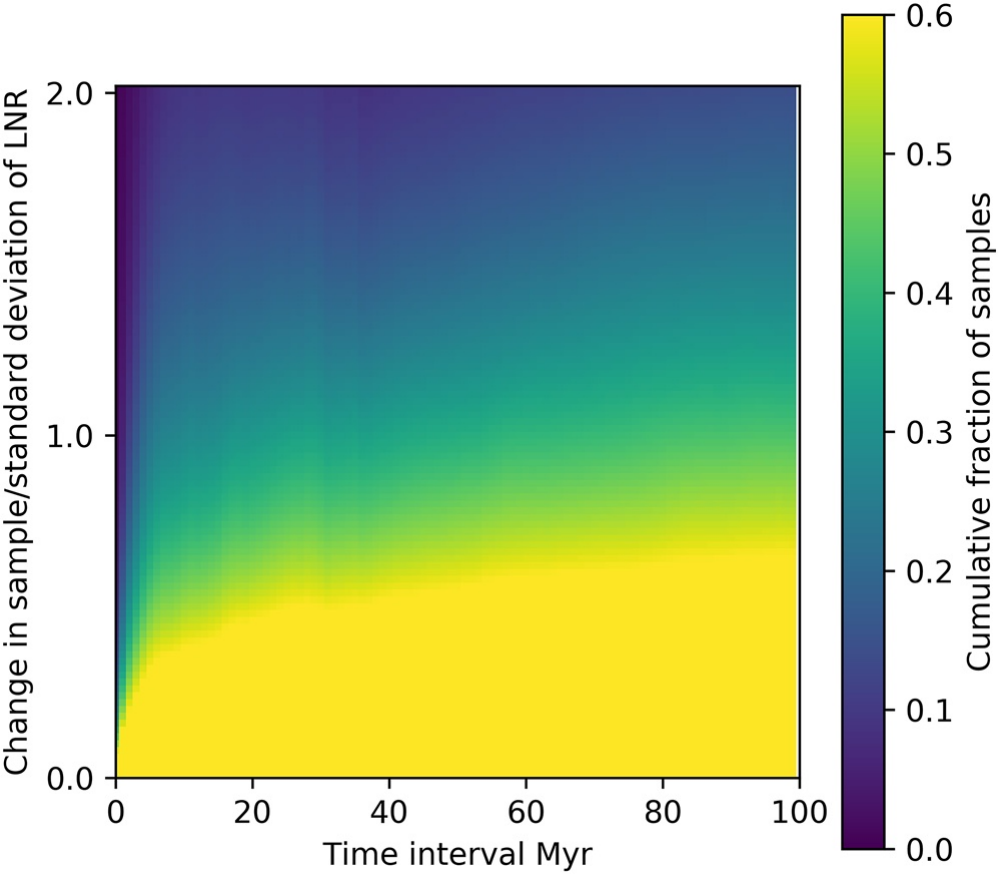
Cumulative histograms of change in NR with time for all frames in all simulations. Change in NR is presented as multiples of the standard deviation of NR within each simulation so that all the results can be presented together.

Whilst there is no clear pattern linking thermal or tectonic structure to LNR, we can at least investigate over what time scales LNR is stable. Figure 6 shows a cumulative histogram of the change in LNR after varying time lapses. When the difference between two random Gaussian signals with a standard deviation of 1 is calculated, 60% of samples would be within approximately 0.75 of each other. Figure 6 suggests that after a time lapse of 100 Myr, the correlation between two measures of LNR is no better than random. With smaller time intervals, the variation between samples is smaller, but there is only a reliable correlation when samples are within 5 Myr of each other.

We also investigate over what timescales we need to sample net rotation in order to produce a Gaussian distribution. This is an important consideration because it determines over what timescales we can reasonably use a Gaussian distribution to model LNR. In order to do this, we separate the LNR for the whole model into chunks of set length. We repeat the exercise, with chunk lengths between 10 samples up to 1,000 samples. We then normalize the LNR in each chunk. If the distribution within the chunk is Gaussian, the normalized chunk distribution will be Gaussian. To test this, we use the Kullback‐Leibler ($D_{KL}$) divergence between the chunk distribution and a Gaussian distribution. If the distribution of LNR with the chunk is Gaussian, the KL divergence will be zero. The KL divergence is calculated by:

(5) $$D_{KL}=\sum P\left(x\right)\log\left(\frac{P\left(x\right)}{Q\left(x\right)}\right)$$

where P(x) and Q(x) are the normalised probability of LNR taking value x in the chunk and a Gaussian distribution respectively. The $D_{KL}$ never reaches 0 because the chunks are too short, so the distribution is imperfectly sampled. However, depending on the model parameters, most models have average $D_{KL}$ over all chunks approaching zero by the time the chunks have length 50 Myr, and within 25 Myr in many cases.

## 4 Discussion

Constraining net rotation of the lithosphere is an important consideration for building absolute plate motion models. There have been several approaches to constraining LNR for the Earth. Most plate motion models produce some LNR. The LNR in these models arises because the modeled plate motions cannot fit geological and palaeomagnetic data with no rotation. Some studies choose to explicitly minimize LNR when fitting data in their plate motion models, for example, Tetley et al. (2019), although this assumption is not constrained by geodynamic considerations, primarily because geodynamic constraints on LNR were not previously available. A key consideration to determining the magnitude and direction of LNR in plate motion models is the choice of mantle reference frame used. Hotspots produced by mantle plumes are often used as a reference frame, with the assumption that the lithosphere moves with respect to quasi‐stable plumes in the mantle. The choice of which plumes to include in the reference frame can alter estimates of net rotation (e.g., Gripp and Gordon (2002) and Doubrovine et al. (2012)). Because we have no reliable record of mantle structure in the geological past and the mantle convection simulations used to study convection evolution often use plate motion models as surface boundary conditions, it is difficult to assess how truly stable competing reference frames are.

Because we are studying convection models in this work, we can sidestep the problem of unstable reference frames. Lateral motion in StagYY is calculated relative to a no net surface rotation reference frame. It is then straightforward to integrate rotation in the mantle to give us LNR in a no mean mantle net rotation reference frame. This is impossible to calculate for the Earth, even at the present day because we have no way of reliably measuring lateral flow for the whole mantle. Perfect knowledge of the modeled LNR allows us to study the variations caused by both convection parameters and the evolution of mantle structures.

Previous studies have identified that lateral viscosity variations are required in geodynamic models to generate lithospheric net rotation, whether introduced through strain and temperature‐dependent viscosity laws or by the addition of highly viscous continents (Hager & O'Connell, 1979; O'Connell et al., 1991; Bercovici et al., 2000). In our models, we find that the only first‐order controls on LNR are choices made in the viscosity law, through the activation energy, activation volume, and presence of a viscosity jump at the 660 km transition zone. In agreement with previous studies, for example, Becker (2006), changes to viscosity in the upper mantle have a much greater effect on LNR, as seen in (Figure 5) where increasing $E_{a}$ strengthens a positive correlation between upper mantle viscosity and LNR. As in Becker (2006) we posit that increased lateral viscosity variations promote surface mobility, allow more lateral motion in the asthenosphere and upper mantle, and potentially weaken the connection between lithosphere and mantle, promoting rotation. The correlation is stronger under the oceans, where more mobile lithosphere leads to a thinner thermal boundary layer and lower viscosity in the upper mantle, allowing more motion between the upper and lower mantle, and therefore more LNR in our models. This can be seen in the positive correlation between the near‐surface temperature and LNR, where a hotter surface is the result of a more mobile surface. We find that the presence of continents has minimal effect on LNR, regardless of how thick they are, unless $E_{a}<3$. This is broadly in agreement with previous studies, which either found continents to have a minimal effect (Gérault et al., 2012), or that continent are important where lateral viscosity variations are low (Becker, 2006; Zhong, 2001). In our models, the continents are passive and do not deform. They reduce the oceanic surface area available for forming ridges and subduction zones by 30% but otherwise do not significantly change the style of oceanic tectonics. In the previous studies where continents were found to make a difference, the viscosity contrast in the mantle was limited to around two orders of magnitude. The continents, therefore, provided the largest viscosity contrast, and slabs were not particularly viscous. In our simulations, at low values of $E_{a}$, the presence or absence of continents make more of a difference, in agreement with the comparable models in the previous studies. However, as soon as $E_{a}$ is increased, the viscosity contrast of the stiff slabs in the mantle has a far greater effect on LNR than the presence or absence of continents. In line with this, we see a weak correlation between increased mid‐mantle viscosity and magnitude of LNR, indicating that periods with more subducting slabs often have higher LNR. However, the correlation is not strong enough to claim that more slabs in the mantle always lead to greater LNR.

Our models are not stochastic, but they are highly non‐linear. LNR is an emergent property of this non‐linear system, where small changes in temperature or rheology at any point in their history can lead to global changes in the mantle structure via many non‐linear interactions. The LNR, therefore, does not arise at random, but the interactions between different parts of the mantle flow which cause LNR are too complicated to be unraveled. We cannot identify any tectonic setting that reliably produces high or low LNR from studying the thermal and viscous structure of the mantle and lithosphere. We see that LNR operates on very different timescales to other parameters. LNR varies much faster than mean temperature and viscosity and does not change in line with changes to surface mobility.

With the relatively small number of models we have for this study, the complexity means that we cannot predict or invert for LNR at any particular point in time. With a much larger number of simulations (probably tens of thousands more), we may be able to use statistical methods, such as machine learning tools, to find characteristics of mantle flow that can determine the magnitude of LNR (Atkins et al., 2016; Gillooly et al., 2019). This is still not particularly practical for absolute plate motion models, because we have limited information on the tectonic arrangement of continents in the geological past. This is likely to limit the possibility of inverting for LNR.

However, despite the non‐linearity at a statistical level, the distribution of net rotation is Gaussian, with the standard deviation dependent on the viscosity of the asthenosphere. The use of asthenospheric anisotropy to estimate the magnitude of LNR for the Earth (as in Conrad & Behn, 2010) therefore seems especially practical. LNR can be modeled as a Gaussian variable in absolute plate motion models or geodynamic simulations. This would allow LNR to vary if other data can be fitted better by using a higher value of LNR, with the knowledge that this is geodynamically reasonable.

We find that LNR is correlated over short time scales, up to about 5 Myr. For recent geological history, we have palaeomagnetic data with sampling rates greater than this, and we should therefore assume that absolute plate motion models using data with resolution below 5 Myr should have smoothly varying LNR. Over longer periods, this means that the jumps seen in models of LNR are not unreasonable and are therefore not necessarily a cause for concern (for a summary of the variability of LNR histories, see Figure 6 in Tetley et al., 2019). We also find that LNR is expected to have an approximately Gaussian distribution with sample lengths as short as 50 Myr.

We performed this study using a 2D spherical annulus geometry. The net rotation is therefore equivalent to that in the plane of a great circle. The velocities of convective motion in a spherical annulus case are generally similar to those produced by a full 3D simulation (Hernlund & Tackley, 2008), therefore we expect the LNR to have similar values. However, we cannot rule out that motion perpendicular to the plane may change the magnitude and mechanisms of net rotation, as the mantle flows around fully 3D slabs and continents. The range of root mean squared values of LNR in our models are generally somewhat lower than those proposed by absolute plate motion studies because we use lower than Earth‐like viscosity variation, apart for models with high $E_{a}$ ($E_{a}$ = 8–12), where we get values comparable to Earth (e.g., simulations 61–63, LNR = 0.18–0.289°/Myr, see Appendix A). That we succeed in producing Earth‐like LNR in 2D model when we use high $E_{a}$ suggests that we adequately model the key features responsible for producing LNR. Having large viscosity contrasts is also clearly a requirement for producing Earth‐like values of LNR. Naturally, because our study was conducted in 2D, we cannot make any inferences about the likely stability of the pole of net rotation.

An interesting feature emerges when comparing the mean and the RMS values of LNR over the runtime of the models, presented in Appendix A. The models generally have mean LNR several orders of magnitude smaller than the RMS. This is expected because the LNR tends to oscillate around 0. This observation, combined with the observation that the correlation length of the models is not much more than 5 Myr may explain some of the mismatches between LNR predicted by absolute plate motion models and present‐day estimates. The model of Torsvik et al. (2010) uses palaeomagnetic data for the last 5 Myr to produce their LNR estimate. Based on our models, this is long enough to begin to average over a large variation in LNR. In contrast, a single present‐day estimate would be a sample from a Gaussian distribution with a standard deviation similar to the RMS values. It is, therefore, possible that both the larger, present‐day values estimates in studies such as Gripp and Gordon (2002) and the smaller, time‐averaged values from plate reconstructions can both be correct. Studies of seismic anisotropy are more likely to produce values closer to the RMS because shearing pre‐deformed olivine in the mantle does not return it to an isotropic state. It, therefore, retains a record of the previous deformation even if the magnitude or direction of LNR changes.

## 5 Conclusion

For the first time, we present lithospheric net rotation measurements from fully self‐consistent convection models with lateral viscosity variations comparable to those expected on Earth. We find that our models can achieve values in the same range as those expected for Earth, although only when the viscosity of the mantle is very temperature sensitive. We, therefore, conclude that the range of values previously published for Earth is geodynamically reasonable. The non‐linearity of mantle convection makes predicting the magnitude and evolution of LNR very difficult, although we do find that increasing the number of slabs in the mid‐mantle is likely, but not guaranteed, to increase LNR. Whilst we cannot predict the evolution of LNR, we do find that it is correlated over short time scales (up to 5 Myr). Beyond this, the distribution of values of LNR is Gaussian, especially if time scales longer than 50 Myr are considered. This provides a new geodynamic constraint on the range of amplitudes and correlation periods expected for Earth, which can be used to constrain future absolute plate motion models.

## Data Availability

A summary of the model data presented in this work is available to download in hdf5 format from Mendeley Data, https://doi.org/10.17632/z3g3zpktsk.1 (Atkins & Coltice, 2021), and includes model parameters, and the time evolution of net rotation, velocity, and viscosity. Figure 4 was produced using GMT 6 (Wessel et al., 2019).

## Appendix A Model Parameters and Resulting Net Rotation

### A.1

Table A1.

**Table A1:** Input Parameters and Selected Convection Metrics for Each Model

Model	Input parameters										Convection metrics				
	$E_{a}$	$V_{a}$	$\Delta \eta^{660}$	σY	$\sigma Y^{cont}$	Cont. size % surf.	$d^{cont}$	heating	$d^{prim}$	$\Delta \eta^{prim}$	$V_{rms}^{surf}$(°/τ)	${LNR}_{rms}$(°/τ)	${LNR}_{rms}$ (°/Myr)	${LNR}_{mean}$(°/Myr)	${LNR}_{rms}$/$V_{rms}^{surf}$
1	1.0	1.0	30.0	2.0e+04	1.0e+08	15:15	0.0692	50.0	0.04	1.0	6.79e+04	2.72e+03	1.42e−02	9.65e−03	4.00e−02
2	1.2	1.0	30.0	2.0e+04	1.0e+08	15:15	0.0692	50.0	0.04	1.0	6.15e+04	1.13e+03	6.38e−03	−4.33e−04	1.84e−02
3	1.0	1.2	30.0	2.0e+04	1.0e+08	15:15	0.0692	50.0	0.04	1.0	6.31e+04	2.24e+03	1.26e−02	7.42e−03	3.54e−02
4	1.2	1.2	30.0	2.0e+04	1.0e+08	15:15	0.0692	50.0	0.04	1.0	6.88e+04	2.70e+03	1.38e−02	7.52e−03	3.92e−02
5	1.5	1.0	30.0	2.0e+04	1.0e+08	15:15	0.0692	50.0	0.04	1.0	6.41e+04	1.78e+03	9.81e−03	−2.87e−03	2.78e−02
6	1.0	1.5	30.0	2.0e+04	1.0e+08	15:15	0.0692	50.0	0.04	1.0	6.10e+04	1.34e+03	7.77e−03	−1.30e−03	2.19e−02
7	2.0	1.0	30.0	2.0e+04	1.0e+08	15:15	0.0692	50.0	0.04	1.0	6.71e+04	2.19e+03	1.12e−02	−1.69e−04	3.26e−02
8	1.0	2.0	30.0	2.0e+04	1.0e+08	15:15	0.0692	50.0	0.04	1.0	6.79e+04	2.81e+03	1.46e−02	−9.51e−03	4.14e−02
9	2.0	2.0	30.0	2.0e+04	1.0e+08	15:15	0.0692	50.0	0.04	1.0	7.00e+04	3.43e+03	1.72e−02	1.18e−03	4.90e−02
10	3.0	1.0	30.0	2.0e+04	1.0e+08	15:15	0.0692	50.0	0.04	1.0	8.78e+04	3.66e+03	1.39e−02	−1.06e−03	4.17e−02
11	3.0	2.0	30.0	2.0e+04	1.0e+08	15:15	0.0692	50.0	0.04	1.0	8.24e+04	4.16e+03	1.75e−02	2.77e−03	5.05e−02
12	3.0	3.0	30.0	2.0e+04	1.0e+08	15:15	0.0692	50.0	0.04	1.0	7.31e+04	5.42e+03	2.57e−02	−8.74e−03	7.42e−02
13	2.0	3.0	30.0	2.0e+04	1.0e+08	15:15	0.0692	50.0	0.04	1.0	6.44e+04	4.60e+03	2.51e−02	2.00e−02	7.15e−02
14	1.0	3.0	30.0	2.0e+04	1.0e+08	15:15	0.0692	50.0	0.04	1.0	5.93e+04	1.89e+03	1.12e−02	−5.05e−03	3.18e−02
15	4.0	3.0	30.0	2.0e+04	1.0e+08	15:15	0.0692	50.0	0.04	1.0	8.41e+04	7.14e+03	2.94e−02	1.48e−03	8.49e−02
16	4.0	2.0	30.0	2.0e+04	1.0e+08	15:15	0.0692	50.0	0.04	1.0	9.66e+04	5.96e+03	2.08e−02	−5.83e−04	6.17e−02
17	4.0	1.0	30.0	2.0e+04	1.0e+08	15:15	0.0692	50.0	0.04	1.0	1.02e+05	5.35e+03	1.70e−02	1.52e−03	5.26e−02
18	3.0	4.0	30.0	2.0e+04	1.0e+08	15:15	0.0692	50.0	0.04	1.0	6.41e+04	6.11e+03	3.32e−02	9.51e−03	9.53e−02
19	2.0	4.0	30.0	2.0e+04	1.0e+08	15:15	0.0692	50.0	0.04	1.0	6.37e+04	4.33e+03	2.36e−02	1.43e−02	6.80e−02
20	1.0	4.0	30.0	2.0e+04	1.0e+08	15:15	0.0692	50.0	0.04	1.0	6.30e+04	3.82e+03	2.16e−02	−1.14e−02	6.06e−02
21	5.0	5.0	30.0	2.0e+04	1.0e+08	15:15	0.0692	50.0	0.04	1.0	7.54e+04	1.66e+04	7.37e−02	3.13e−03	2.21e−01
22	4.0	4.0	30.0	2.0e+04	1.0e+08	15:15	0.0692	50.0	0.04	1.0	7.80e+04	9.45e+03	4.17e−02	−3.51e−03	1.21e−01
23	3.0	5.0	30.0	2.0e+04	1.0e+08	15:15	0.0692	50.0	0.04	1.0	7.21e+04	7.61e+03	3.65e−02	5.82e−04	1.06e−01
24	1.0	1.0	30.0	2.0e+04	2.0e+06	15:15	0.0692	50.0	0.04	1.0	6.45e+04	3.61e+03	1.99e−02	1.78e−02	5.61e−02
25	4.0	4.0	1.0	2.0e+04	1.0e+08	15:15	0.0692	50.0	0.04	1.0	3.03e+04	1.96e+03	2.08e−02	3.90e−04	6.47e−02
26	4.0	4.0	1.0	2.0e+04	1.0e+08	15:15	0.0692	50.0	0.10	1.0	2.91e+04	1.77e+03	1.95e−02	1.43e−03	6.07e−02
27	4.0	4.0	30.0	2.0e+04	1.0e+08	15:15	0.0692	50.0	0.10	1.0	7.65e+04	8.95e+03	4.03e−02	−5.07e−03	1.17e−01
28	4.0	4.0	30.0	2.0e+04	1.0e+08	15:15	0.0692	50.0	0.15	1.0	7.56e+04	8.93e+03	4.07e−02	−1.64e−03	1.18e−01
29	4.0	4.0	30.0	2.0e+04	1.0e+08	15:15	0.0692	50.0	0.25	1.0	7.47e+04	8.94e+03	4.14e−02	−1.05e−03	1.20e−01
30	1.0	1.0	1.0	2.0e+04	1.0e+08	15:15	0.0692	50.0	0.04	1.0	1.76e+04	1.89e+02	3.78e−03	−1.98e−03	1.07e−02
31	4.0	4.0	1.0	2.0e+04	1.0e+08	15:15	0.0692	50.0	0.10	1.0	2.92e+04	1.78e+03	1.97e−02	2.44e−03	6.11e−02
32	4.0	4.0	1.0	2.0e+04	1.0e+08	15:15	0.0692	50.0	0.15	1.0	3.00e+04	1.84e+03	1.96e−02	−2.20e−03	6.14e−02
33	0.0	0.0	30.0	2.0e+04	1.0e+08	15:15	0.0692	50.0	0.04	1.0	1.01e+05	8.84e+01	3.13e−04	1.14e−05	8.79e−04
34	0.0	0.0	1.0	2.0e+04	1.0e+08	15:15	0.0692	50.0	0.04	1.0	1.40e+04	4.01e+01	1.02e−03	1.07e−04	2.86e−03
35	0.0	1.0	1.0	2.0e+04	1.0e+08	15:15	0.0692	50.0	0.04	1.0	1.33e+04	4.86e+01	1.31e−03	−2.58e−05	3.66e−03
36	1.0	0.0	1.0	2.0e+04	1.0e+08	15:15	0.0692	50.0	0.04	1.0	1.74e+04	1.41e+02	2.82e−03	5.91e−04	8.12e−03
37	1.0	0.0	30.0	2.0e+04	1.0e+08	15:15	0.0692	50.0	0.04	1.0	6.93e+04	2.11e+03	1.07e−02	−5.88e−03	3.04e−02
38	0.0	1.0	30.0	2.0e+04	1.0e+08	15:15	0.0692	50.0	0.04	1.0	8.98e+04	1.25e+02	5.02e−04	−1.21e−05	1.39e−03
39	0.0	8.0	30.0	2.0e+04	1.0e+08	15:15	0.0692	50.0	0.04	1.0	3.21e+04	1.21e+02	1.35e−03	−5.72e−04	3.76e−03
40	0.0	0.0	30.0	2.0e+04	1.0e+08	15:15	0.0692	25.0	0.04	1.0	9.76e+04	7.37e+01	2.71e−04	−5.22e−06	7.55e−04
41	0.0	0.0	30.0	2.0e+04	1.0e+08	15:15	0.0692	100.0	0.04	1.0	1.00e+05	9.87e+01	3.52e−04	−1.19e−05	9.86e−04
42	0.0	0.0	30.0	2.0e+02	1.0e+08	15:15	0.0692	100.0	0.04	1.0	1.35e+05	3.65e+02	9.67e−04	−3.21e−05	2.70e−03
43	0.0	0.0	30.0	2.0e+06	1.0e+08	15:15	0.0692	100.0	0.04	1.0	9.53e+04	2.74e+00	1.03e−05	7.11e−07	2.88e−05
44	4.0	4.0	30.0	2.0e+04	1.0e+08	15:15	0.0692	100.0	0.04	1.0	1.79e+05	1.10e+04	2.08e−02	8.84e−03	6.17e−02
45	4.0	4.0	30.0	2.0e+04	1.0e+08	15:15	0.0692	25.0	0.04	1.0	3.83e+04	1.01e+04	9.08e−02	4.03e−03	2.64e−01
46	4.0	4.0	1.0	2.0e+04	1.0e+08	15:15	0.0692	25.0	0.04	1.0	1.75e+04	2.61e+03	5.09e−02	−2.39e−03	1.49e−01
47	2.0	2.0	30.0	2.0e+02	1.0e+08	15:15	0.0692	50.0	0.04	1.0	7.44e+04	3.46e+03	1.65e−02	−6.12e−03	4.64e−02
48	2.0	2.0	30.0	2.0e+06	1.0e+08	15:15	0.0692	50.0	0.04	1.0	4.16e+04	1.11e+04	9.43e−02	8.60e−02	2.67e−01
49	0.0	0.0	30.0	2.0e+02	1.0e+08	15:15	0.0692	25.0	0.04	1.0	1.08e+05	2.00e+02	6.63e−04	−8.09e−06	1.85e−03
50	0.0	0.0	30.0	2.0e+06	1.0e+08	15:15	0.0692	25.0	0.04	1.0	1.06e+05	2.21e+00	7.50e−06	−1.34e−08	2.09e−05
51	0.0	4.0	30.0	2.0e+04	1.0e+08	15:15	0.0692	50.0	0.04	1.0	4.57e+04	3.40e+02	2.68e−03	−6.60e−04	7.44e−03
52	2.0	2.0	1.0	2.0e+06	1.0e+08	15:15	0.0692	50.0	0.04	1.0	3.91e+02	2.59e+02	2.34e−01	1.44e−02	6.63e−01
53	2.0	2.0	1.0	2.0e+02	1.0e+08	15:15	0.0692	50.0	0.04	1.0	2.93e+04	5.58e+02	6.82e−03	3.41e−03	1.90e−02
54	2.0	2.0	30.0	2.0e+04	1.0e+08	10:20	0.0692	50.0	0.04	1.0	6.94e+04	4.26e+03	2.15e−02	1.76e−02	6.13e−02
55	2.0	2.0	30.0	2.0e+04	1.0e+08	6:24	0.0692	50.0	0.04	1.0	6.99e+04	4.14e+03	2.08e−02	1.70e−02	5.92e−02
56	2.0	2.0	1.0	2.0e+04	1.0e+08	6:24	0.0692	50.0	0.04	1.0	2.15e+04	3.46e+02	5.54e−03	−3.80e−04	1.61e−02
57	1.0	1.0	30.0	2.0e+04	1.0e+08	0:0	0.0000	50.0	0.04	1.0	5.08e+04	5.35e+02	3.72e−03	−3.93e−04	1.05e−02
58	1.0	1.0	1.0	2.0e+04	1.0e+08	0:0	0.0000	50.0	0.04	1.0	1.51e+04	6.62e+01	1.54e−03	−1.03e−04	4.38e−03
59	4.0	4.0	30.0	2.0e+04	1.0e+08	0:0	0.0000	50.0	0.04	1.0	7.87e+04	1.51e+04	6.58e−02	3.32e−03	1.92e−01
60	4.0	4.0	1.0	2.0e+04	1.0e+08	0:0	0.0000	50.0	0.04	1.0	2.92e+04	2.57e+03	2.94e−02	2.93e−03	8.80e−02
61	8.0	8.0	30.0	2.0e+04	1.0e+08	0:0	0.0000	50.0	0.04	1.0	9.62e+04	5.14e+04	1.82e−01	2.19e−03	5.35e−01
62	8.0	8.0	1.0	2.0e+04	1.0e+08	0:0	0.0000	50.0	0.04	1.0	5.85e+04	2.31e+04	1.31e−01	−1.68e−02	3.95e−01
63	12.0	12.0	1.0	2.0e+04	1.0e+08	15:15	0.0692	50.0	0.04	1.0	3.01e+04	4.77e+04	2.85e−01	−3.27e−02	1.58e+00
64	1.0	1.0	30.0	2.0e+04	1.0e+08	15:15	0.1384	50.0	0.04	1.0	6.94e+04	3.90e+03	2.00e−02	−1.69e−02	5.62e−02
65	1.0	1.0	1.0	2.0e+04	1.0e+08	15:15	0.1384	50.0	0.04	1.0	1.72e+04	1.70e+02	3.48e−03	1.02e−03	9.87e−03
66	1.0	12.0	30.0	2.0e+04	1.0e+08	15:15	0.0692	50.0	0.04	1.0	2.66e+04	3.14e+03	4.22e−02	−3.00e−02	1.18e−01
67	4.0	4.0	30.0	2.0e+04	1.0e+08	15:15	0.1384	50.0	0.04	1.0	7.90e+04	9.31e+03	4.10e−02	−1.64e−03	1.18e−01
68	4.0	4.0	1.0	2.0e+04	1.0e+08	15:15	0.1384	50.0	0.04	1.0	2.99e+04	1.88e+03	1.99e−02	−2.59e−04	6.29e−02
69	4.0	1.0	30.0	2.0e+04	1.0e+08	15:15	0.1384	50.0	0.04	1.0	1.03e+05	6.44e+03	2.03e−02	−3.85e−03	6.26e−02
70	1.0	4.0	30.0	2.0e+04	1.0e+08	15:15	0.1384	50.0	0.04	1.0	5.63e+04	3.32e+03	2.09e−02	1.00e−02	5.90e−02
71	4.0	4.0	30.0	2.0e+04	1.0e+08	15:15	0.2076	50.0	0.04	1.0	7.51e+04	8.76e+03	4.05e−02	−1.71e−03	1.17e−01
72	4.0	4.0	1.0	2.0e+04	1.0e+08	15:15	0.2076	50.0	0.04	1.0	2.95e+04	1.78e+03	1.92e−02	−9.24e−04	6.02e−02
73	1.0	1.0	30.0	2.0e+04	1.0e+08	15:15	0.2076	50.0	0.04	1.0	6.12e+04	3.00e+03	1.74e−02	1.32e−02	4.91e−02
74	1.0	1.0	1.0	2.0e+04	1.0e+08	15:15	0.2076	50.0	0.04	1.0	1.73e+04	1.57e+02	3.20e−03	−5.40e−04	9.07e−03
75	4.0	4.0	30.0	2.0e+02	1.0e+08	15:15	0.2076	50.0	0.04	1.0	6.18e+04	1.44e+04	8.18e−02	−5.06e−02	2.33e−01
76	4.0	4.0	1.0	2.0e+02	1.0e+08	15:15	0.2076	50.0	0.04	1.0	3.14e+04	1.90e+03	2.15e−02	1.34e−02	6.03e−02
77	0.0	0.0	1.0	2.0e+04	1.0e+08	0:0	0.0000	50.0	0.04	1.0	1.39e+04	4.00e+01	1.03e−03	6.69e−05	2.89e−03
78	0.0	0.0	30.0	2.0e+04	1.0e+08	0:0	0.0000	50.0	0.04	1.0	8.99e+04	7.29e+01	2.89e−04	−2.22e−07	8.11e−04
79	0.0	0.0	30.0	2.0e+04	1.0e+08	15:15	0.1384	50.0	0.04	1.0	9.43e+04	7.49e+01	2.84e−04	−2.59e−05	7.95e−04
80	1.0	4.0	30.0	2.0e+04	1.0e+08	0:0	0.0000	50.0	0.04	1.0	5.57e+04	1.09e+03	7.02e−03	−5.90e−04	1.95e−02
81	4.0	1.0	30.0	2.0e+04	1.0e+08	0:0	0.0000	50.0	0.04	1.0	8.80e+04	4.26e+03	1.57e−02	3.28e−04	4.84e−02
82	2.0	2.0	1.0	2.0e+04	1.0e+08	0:0	0.0000	50.0	0.04	1.0	1.92e+04	2.94e+02	5.28e−03	8.78e−05	1.53e−02
83	4.0	1.0	1.0	2.0e+04	1.0e+08	0:0	0.0000	50.0	0.04	1.0	3.69e+04	1.53e+03	1.13e−02	−1.56e−03	4.14e−02
84	1.0	4.0	1.0	2.0e+04	1.0e+08	0:0	0.0000	50.0	0.04	1.0	1.14e+04	1.56e+02	4.85e−03	−1.06e−03	1.37e−02
85	1.0	4.0	1.0	2.0e+04	1.0e+08	15:15	0.0692	50.0	0.04	1.0	1.60e+04	2.17e+02	4.75e−03	8.95e−04	1.36e−02
86	4.0	1.0	1.0	2.0e+04	1.0e+08	15:15	0.0692	50.0	0.04	1.0	3.78e+04	1.25e+03	9.03e−03	−7.17e−04	3.31e−02
87	2.0	2.0	30.0	2.0e+04	1.0e+08	0:0	0.0000	50.0	0.04	1.0	4.86e+04	1.50e+03	1.08e−02	6.47e−04	3.08e−02
88	6.0	6.0	30.0	2.0e+04	1.0e+08	0:0	0.0000	50.0	0.04	1.0	7.93e+04	3.78e+04	1.67e−01	−9.84e−03	4.77e−01
89	6.0	6.0	30.0	2.0e+04	1.0e+08	15:15	0.0692	50.0	0.04	1.0	8.81e+04	2.41e+04	9.18e−02	−3.20e−04	2.74e−01
90	6.0	6.0	1.0	2.0e+04	1.0e+08	0:0	0.0000	50.0	0.04	1.0	4.20e+04	8.06e+03	6.27e−02	−2.11e−02	1.92e−01
91	6.0	6.0	1.0	2.0e+04	1.0e+08	15:15	0.0692	50.0	0.04	1.0	4.57e+04	6.05e+03	4.00e−02	−2.75e−04	1.32e−01
92	3.0	3.0	30.0	2.0e+04	1.0e+08	0:0	0.0000	50.0	0.04	1.0	5.64e+04	4.42e+03	2.76e−02	−4.60e−03	7.83e−02
93	3.0	3.0	1.0	2.0e+04	1.0e+08	0:0	0.0000	50.0	0.04	1.0	2.28e+04	1.17e+03	1.72e−02	−2.78e−03	5.14e−02
94	3.0	3.0	1.0	2.0e+04	1.0e+08	15:15	0.0692	50.0	0.04	1.0	2.39e+04	9.11e+02	1.23e−02	1.45e−04	3.80e−02
95	3.0	3.0	1.0	2.0e+02	1.0e+08	0:0	0.0000	50.0	0.04	1.0	2.34e+04	7.66e+02	1.18e−02	7.79e−04	3.28e−02
96	3.0	3.0	1.0	2.0e+03	1.0e+08	0:0	0.0000	50.0	0.04	1.0	2.08e+04	8.00e+02	1.37e−02	−1.76e−04	3.85e−02
97	2.0	2.0	1.0	2.0e+03	1.0e+08	0:0	0.0000	50.0	0.04	1.0	1.94e+04	4.07e+02	7.47e−03	4.97e−04	2.10e−02
98	2.0	2.0	1.0	2.0e+02	1.0e+08	0:0	0.0000	50.0	0.04	1.0	2.06e+04	4.44e+02	7.70e−03	4.88e−04	2.15e−02
99	2.0	2.0	30.0	2.0e+02	1.0e+08	0:0	0.0000	50.0	0.04	1.0	6.15e+04	2.38e+03	1.39e−02	−8.31e−04	3.87e−02
100	2.0	2.0	30.0	2.0e+03	1.0e+08	0:0	0.0000	50.0	0.04	1.0	7.37e+04	2.28e+03	1.11e−02	−1.26e−03	3.10e−02
101	3.0	3.0	30.0	2.0e+02	1.0e+08	0:0	0.0000	50.0	0.04	1.0	5.16e+04	2.74e+03	1.91e−02	−1.96e−03	5.31e−02
102	3.0	3.0	30.0	2.0e+03	1.0e+08	0:0	0.0000	50.0	0.04	1.0	5.39e+04	3.72e+03	2.48e−02	2.27e−03	6.90e−02
103	3.0	3.0	30.0	2.0e+04	1.0e+08	15:15	0.0692	50.0	0.04	10.0	7.44e+04	5.43e+03	2.51e−02	−7.93e−03	7.31e−02
104	3.0	3.0	30.0	2.0e+04	1.0e+08	15:15	0.0692	50.0	0.08	10.0	7.17e+04	4.82e+03	2.34e−02	9.41e−03	6.72e−02
105	3.0	3.0	30.0	2.0e+04	1.0e+08	15:15	0.0692	50.0	0.04	2.0	7.04e+04	4.52e+03	2.23e−02	6.01e−03	6.42e−02
106	3.0	3.0	30.0	2.0e+04	1.0e+08	15:15	0.0692	50.0	0.08	2.0	7.24e+04	4.98e+03	2.40e−02	7.52e−03	6.88e−02
107	3.0	3.0	1.0	2.0e+04	1.0e+08	15:15	0.0692	50.0	0.04	2.0	2.39e+04	9.72e+02	1.33e−02	−5.38e−04	4.06e−02
108	3.0	3.0	30.0	1.0e+04	1.0e+08	0:0	0.0000	50.0	0.04	1.0	6.58e+04	5.13e+03	2.76e−02	−1.47e−03	7.79e−02
109	3.0	3.0	1.0	1.0e+04	1.0e+08	0:0	0.0000	50.0	0.04	1.0	2.14e+04	7.91e+02	1.32e−02	−1.74e−03	3.70e−02
110	4.0	4.0	30.0	2.0e+03	1.0e+08	0:0	0.0000	50.0	0.04	1.0	5.08e+04	5.30e+03	3.74e−02	−3.57e−03	1.04e−01

*Note*. $E_{a}$ viscosity activation energy; $V_{a}$ viscosity activation volume; $\Delta \eta^{660}$ transition zone viscosity jump factor; σY oceanic yield stress; $\sigma Y^{cont}$ continental margin; continent size for two continents as percentage of surface area; $d^{cont}$ continent thickness; internal radiogenic heating; $d^{prim}$ thickness of initial primordial layer at base of mantle; $\Delta \eta^{prim}$ viscosity factor for primordial material; $V_{rms}^{surf}$ non‐dimensional root mean squared surface velocity; ${LNR}_{rms}$ non‐dimensional and dimensionalised root mean squared net rotation; ${LNR}_{mean}$ mean dimensionalised LNR; ${LNR}_{rms}$/$V_{rms}^{surf}$ LNR scaled by RMS surface velocity.
